# A Systematic Review of the Complex Effects of Cannabinoids on Cerebral and Peripheral Circulation in Animal Models

**DOI:** 10.3389/fphys.2018.00622

**Published:** 2018-05-29

**Authors:** J. Sebastian Richter, Véronique Quenardelle, Olivier Rouyer, Jean Sébastien Raul, Rémy Beaujeux, Bernard Gény, Valérie Wolff

**Affiliations:** ^1^Department of Interventional Neuroradiology, University Hospital of Strasbourg, Strasbourg, France; ^2^Institute of Image-Guided Surgery (IHU), Strasbourg, France; ^3^Equipe d'Accueil 3072, University of Strasbourg, Strasbourg, France; ^4^Stroke Unit, University Hospital, Strasbourg, France; ^5^Department of Physiology and Functional Explorations, University Hospital of Strasbourg, Strasbourg, France; ^6^Institute of Legal Medicine, University of Strasbourg, Strasbourg, France

**Keywords:** cannabis, cannabinoids, vasoreactivity, vasoconstriction, cerebral vasospasm, animal models, stroke

## Abstract

While cannabis is perceived as a relatively safe drug by the public, accumulating clinical data suggest detrimental cardiovascular effects of cannabinoids. Cannabis has been legalized in several countries and jurisdictions recently. Experimental studies specifically targeting cannabinoids' effects on the cerebral vasculature are rare. There is evidence for transient vasoconstrictive effects of cannabinoids in the peripheral and cerebral vasculature in a complex interplay of vasodilation and vasoconstriction. Vasoreactivity to cannabinoids is dependent on the specific molecules, their metabolites and dose, baseline vascular tone, and vessel characteristics as well as experimental conditions and animal species. We systematically review the currently available literature of experimental results in *in vivo* and *in vitro* animal studies, examining cannabinoids' effects on circulation and reactive vasodilation or vasoconstriction, with a particular focus on the cerebral vascular bed.

## Introduction

Of the estimated 203 million cannabis users worldwide and 14.6 million young European consumers (age 15–34), more than 13 million are dependent on cannabis (Degenhardt and Hall, 2012). These numbers have been increasing during the first decade of the current century, making cannabis by far the most commonly used illicit drug of our time (WHO, 2016). Legalization of marijuana in several countries and states of the United States for therapeutic and recreational use has reignited a worldwide discussion about risks and benefits of its consumption. Publicly, cannabis has been perceived as a relatively “safe drug” by many. For others, adverse effects of cannabinoid consumption has not been appreciated sufficiently (Volkow et al., 2014; Rose, 2016). A recent review of the cardiovascular risk of using cannabis has highlighted the controversy between its therapeutic and its adverse effects (Goyal et al., 2017).

Cannabis' risks are particularly apparent in young populations, in which consumption is highest. Cannabis inhalation was linked to cerebral ischemic infarction as early as 40 years ago, before sophisticated cerebral imaging techniques were available to confirm clinical findings (Garrett et al., 1977). It has been observed that strokes occurred after inhalation of particularly large doses of cannabis (prolonged intense consumption, exact doses unspecified) and that the subjected patients were young (Cooles and Michaud, 1987). Numerous and more recent case reports and reviews have highlighted the correlation of cannabis (herbal form or resin) and synthetic cannabinoid consumption (“spice,” e.g., the synthetic molecule ADB-FUBINACA) to ischemic and hemorrhagic cerebrovascular events (Rose et al., 2015; Rumalla et al., 2016a,b; El Mesbahy et al., 2017; Moeller et al., 2017; Volpon et al., 2017; Wolff and Jouanjus, 2017). This clinical evidence clearly suggests a role of cannabis in the etiology of cerebral stroke (Wolff et al., 2015c).

**Figure 1:**
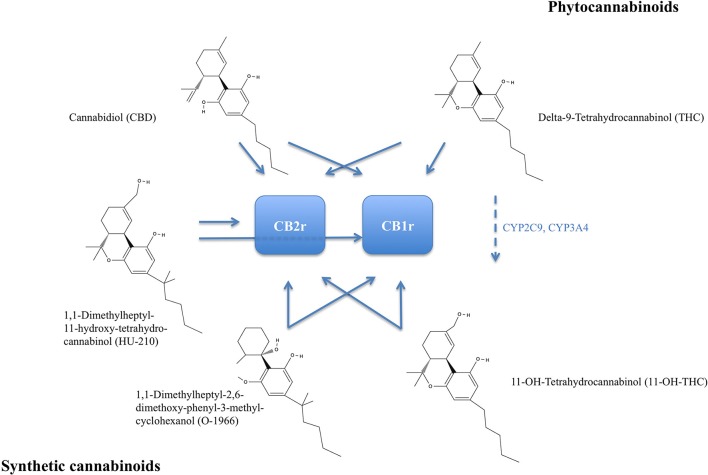
Chemical structures of major phytocannabinoids: Delta-9-Tetrahydrocannabinol (THC) and Cannabidiol. Delta-9-THC is metabolized to 11-OH-Tetrahydrocannabinol by cytochrome P450 2C9 (CYP2C9) and cytochrome P450 3A4 (CYP3A4), broken arrow. Synthetic analogs 1,1-Dimethylheptyl-11-hydroxy-tetrahydro-cannabinol (HU-210) and 1,1-Dimethylheptyl-2,6-dimethoxy-phenyl-3-methyl-cyclohexanol (O-1966). Receptor affinity indicated by blue arrows. THC and 11-OH-THC are psychoactive and both more selectively binding to the CB1 receptor (CB1r). CB2r, CB2 receptor.

The degree of beneficial and deleterious effects of cannabis is generally linked to its potency, which in turn depends on the content of cannabis' psychoactive component Tetrahydrocannabinol (THC). In many European countries, Oceania, and the United States, THC content in consumed cannabis probes has been reported to increase (UN, 2015). Cannabinoids (CBs) comprise a group of chemical compounds that have varying affinity to the type 1 CB (CB1) receptor and type 2 CB (CB2) receptor. CB receptors belong to the family of G-protein coupled receptors (GPCR) with predominantly inhibitory function on the cyclic adenosine monophosphate (cAMP) pathway in intracellular signal transduction. Cannabinoids can generally be classified into phytocannabinoids, mainly isolated from *Cannabis sativa*, endocannabinoids, and synthetic cannabinoids (overview of major phytocannabinoids, synthetic analog, and action on CB receptors in Figure 1).

**Figure 2:**
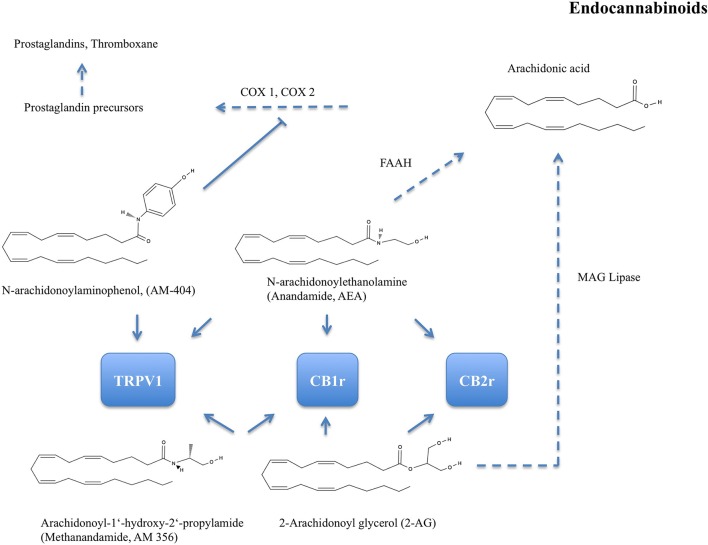
Chemical structures of major endocannabinoids: N-arachidonoylethanolamine (Anandamide or AEA), 2-Arachidonoyl glycerol (2-AG), and N-arachidonoylaminophenol (AM-404). One of their synthetic analogs is Arachidonoyl-1'hydroxy-2'-propylamide (Methanandamide or AM-356). AEA is hydrolyzed by fatty acid amino hydrolase (FAAH) to 5,8,11,14-Eicosatetraenoic acid (arachidonic acid). 2-AG is hydrolyzed by monoacylglycerol (MAG) lipase to arachidonic acid. Arachidonic acid is metabolised via prostaglandin precursors to vasodilative prostaglandins and thromboxane by cyclo-oxygenase (COX-) 1 and 2. AM-404 is an endogenous ligand to transient receptor potential cation-channel subfamily V member 1 (TRPV1). AM-404 inhibits COX-1 and COX-2 and thus attenuates prostaglandin synthesis. AEA, 2-AG, and AM-356 are selective CB1 receptor (CB1r) agonists. CB2r, CB2 receptor.

As one of the first phytocannabinoids, Δ9-THC was isolated by Gaoni and Mechoulam (1964). In *C. sativa* the molecule is present mainly in its acidic form, as tetrahydrocannabinolic acid, then decarboxylated to THC. The lipophilic molecule THC has an affinity to adipose tissues and it is known to exert antioxidant activity (Lastres-Becker et al., 2005). Endocannabinoids have been identified as endogenous ligands to CB1 and CB2 receptors (overview of major endocannabinoids, a synthetic analog, and target receptors in Figure 2). Together with the involved metabolic enzymes, they constitute the endocannabinoid system (Maccarrone et al., 2015). The first endocannabinoid, the arachidonic acid derivative arachidonylethanolamide (AEA or anandamide), was isolated and identified from porcine brains (Devane et al., 1992).

Both CB receptors have been localized at the cell surface of various tissues, but in the central and peripheral nervous system CB1 receptors are expressed more densely (Herkenham et al., 1990; Matsuda et al., 1990; Onaivi, 2006). CB1 receptors are also expressed in endothelial cells and on mitochondrial membranes (Liu et al., 2000; Harkany and Horvath, 2017). Their inhibitory action on cAMP production is mediated by activation of the adenylyl cyclase inhibitor subunit of G proteins (G_*i*/*o*_ proteins). This results in an inhibition of N- and P/Q-type calcium currents and an activation of A-type, inwardly rectifying potassium currents and mitogen-activated protein kinase (Sierra et al., 2018). Other binding sites for the endocannabinoid AEA and the phytocannabinoid Δ9-THC include the transient receptor potential cation-channel subfamily V member 1 (TRPV1) and peroxisome proliferator-activator receptors (PPARs). The latter belong to the group of ligand-activated transcription factors. Δ9-THC also acts as an agonist on the GPCR 18 (GPR18 or N-Arachidonyl glycine receptor, NAGly receptor), along with endocannabinoids, such as the abnormal cannabidiol (Abn-CBD) and NAGly, a metabolite of AEA (Kohno et al., 2006). GPR18 has been proposed as a potential cannabinoid receptor (Console-Bram et al., 2014).

Many details of the cannabinoids-induced pathophysiological processes of the cardiovascular system remain poorly understood. One approach to better understanding is to study the vascular effects of cannabinoids in animals. Recent clinical observations suggest a correlation of cannabis use with the incidence of ischemic stroke in the young. It was our objective to systematically review the currently available literature on the effects of cannabinoids on circulation in experimental animal models, with a particular focus on the cerebral vascular bed.

## Methods

### Literature search and inclusion criteria

We conducted a systematic literature search via the PubMed and Cochrane platforms on September 1st, 2017, on all articles published and indexed up to this point. To identify relevant articles on original research, we associated terms referring to the use of cannabis or cannabis-related psychoactive substances, animal models, and vascular reactivity or stroke according to a predefined search algorithm (see below). All experimental studies on animals were eligible. We did not place any restrictions on the design of the study to be included, as long as it was conducted on an animal model. Personal databases of the authors were screened for relevant articles, as were bibliography lists of relevant reviews, which were then manually added.

### Search algorithm

(THC OR marijuana OR cannabis OR cannabinoids OR hashish) and (animal OR animal model OR ape OR monkey OR primate OR pig OR swine OR porc OR porcine OR dog OR cat OR sheep OR rabbit OR guinea pig OR rat OR mouse) and (stroke OR vasospasm OR vasoconstriction OR vasodilation OR reversible cerebral vasoconstriction syndrome)

### Exclusion criteria

Articles were excluded that pertained to humans or human clinical case reports, clinical case series, and cytological studies, or when they focused on non-vascular, neuroprotective effects of cannabinoids. Letters, editorials, and comments were excluded after checking for cross-references that were not identified by our research, as well as articles that were written in languages other than English, German, or French.

### Screening process

After application of the inclusion criteria, duplicates were removed and all remaining articles were screened manually. Articles were also screened for references not generated by our research which were then added to the full-text assessment.

## Results

### Literature search

Our systematic literature search revealed 380 publications on PubMed. To these, 20 articles were added manually from personal databases (J.S.R. and V.W.). After screening of titles and abstracts, 19 articles were added from cross-references and in total 62 articles remained for full-text assessment. Of these, 11 articles were excluded with reason, leaving included 51 articles for qualitative synthesis (see Figure S1 in Supplementary Material).

### Vascular effects of cannabinoids in rodents

#### Differential vasoreactivity in cerebral vasculature

In the late 90s, exploration of the cerebral distribution of CB receptors began after it had been proposed to be particularly heterogenous. Neuronal activity was visualized by autoradiography of the changes to local blood flow after intravenous (i.v.) injection of Δ-9-THC by Bloom et al. (1997). The authors analyzed 37 regions, distributed in mesocorticolimbic, neocortical, hypothalamic, and nigrostriatal regions, as well as the cerebellum and white matter. Intriguingly, blood flow was decreased in the CA1 region of the hippocampus, frontal and medial prefrontal cortex, the nucleus accumbens, and the claustrum. It was unaltered in the medial septum, ventral tegmental area, caudate, temporal, parietal and occipital cortex, and the cerebellum. In total, local cerebral blood flow (CBF) was affected in 16 out of 37 measured areas. CB receptor-mediated regulation on CBF was confirmed by more autoradiographic measurements showing blood flow reduction in the amygdala, cingulate, frontal, prepyriform, sensorimotor, and claustrocortex at a dose of 3.0 mg/kg AEA. Further blood flow depression occurred in the CA1 and CA2 regions of the hippocampus, the rostral core portion of the nucleus accumbens, and the rostral caudate nucleus at a dose of 30.0 mg/kg AEA (i.v.) in conscious rats (Stein et al., 1998). The variation was interpreted as a secondary effect to decreased neuronal activity. In isolated middle cerebral arteries of rats, the endogenous CB1 receptor-agonist 2-AG was shown to attenuate vasoconstriction by the thromboxane mimetic U-46619 (Hillard et al., 2007). More recently, Iring et al. (2013) did not find any significant influence of CB1 receptor activity on the regulation of cerebrocortical circulation under resting conditions in normotensive rats when activating endogenous AEA. There was, however, an indication of CB1 receptor-independent systemic hypertension as a result to enhancement of endocannabinoids. An increase of CBF was measured by laser-Doppler velocimetry of cerebrocortical blood flow (CoBF) and some evidence suggested an impact of the CB1 receptor on cerebral circulation autoregulation (Iring et al., 2013). Abnormal cannabidiol (Abn-CBD), an endogenous agonist to GPR18 and the novel endothelial receptor CB_*E*_, devoid of action on CB1 and CB2 receptors, inhibited endothelin-induced vasoconstriction endothelium-dependently by a mechanism involving SK_*Ca*_ channels in retinal arterioles (MacIntyre et al., 2014). In a mouse model, it was shown that the combination of a CB1 receptor-antagonist with a CB2 receptor-agonist could increase CBF during an induced ischemia and in this manner act neuroprotectively (Zhang et al., 2008).

#### Vasodilation in peripheral vasculature

In urethane-anesthetized animals monitored by a jugular catheter for blood pressure measurements, Graham and Li (1973) revealed a drop in systemic blood pressure and pulse as well as respiratory rate of a duration of up to 60 min after i.v.-injection of cannabis-extract (10 mg/kg). The reduction in blood pressure, pulse and respiratory rate was dose-dependent. It was attenuated by an induction of tolerance by intraperitoneal pretreatment over the course of 14 days (50 mg/kg per day). It was also attenuated, but still detectable, when cannabis extract was replaced by Δ-1- or Δ-6-THC. The effect was not reproducible with cannabinol or cannabidiol as the active agent.

A delayed hypotensive response followed a short-lasting vasoconstrictor response in reaction to Δ-8- and Δ-9-THC infusion into perfused hindquarters (i.v. and intra-arterial, i.a., administration) of the rat (Adams et al., 1976). Kosersky (1978) illustrated that cannabinoids' action on the vasculature may depend on other factors than the substance itself. Orally administered Δ-9-THC did not provoke hypotension in normotensive specimen, but only in spontaneously hypertensive rats. This hinted to a partial dependence of the substance's effect on baseline blood pressure. A tolerance effect following pre-treatment was not observed in contrast to Graham's and Li's reports on cannabis extract.

#### The triple effect, an early indication of reactive vasoconstriction in peripheral vascular beds

**Figure 3:**
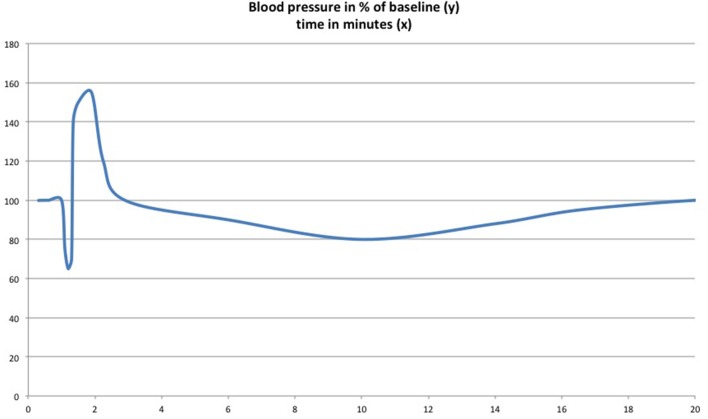
The “triple effect” or “triphasic effect.” Upon injection of Δ-9-THC (Siqueira et al., 1979) and anandamide (Varga et al., 1996) in urethane-anesthetized rats, an initial drop in blood pressure is followed by a short-lasting blood pressure peak mediated by vasoconstriction and then by delayed moderate and transient hypotension. Scheme adapted from the results of the aforementioned publications.

Siqueira et al. (1979) first observed what they called a triple effect following i.v. Δ-9-THC injection (2–10 mg/kg) in rats, when they monitored the animals blood pressure: (1) short-lasting immediate hypotension mediated by the vagal nerve, (2) a vasoconstriction-related rise in blood-pressure within a 30 s delay, and (3) a final persistent hypotension, supposedly by a central decrease in sympathetic tone. The effect on vagal tone was confirmed shortly thereafter with Δ-9-THC in freely moving animals (Kawasaki et al., 1980). A dose-dependency was demonstrated for the hypertensive reaction. Medium doses of 6 mg/kg provoked a more marked hypertension than both lower (1 mg/kg) and higher (8 mg/kg) concentrated injections. This was the first study on non-anesthetized animals. The triple effect was again observed as a reaction to CB1 receptor-agonist AEA i.v.-injection (Varga et al., 1996). Describing the previously mentioned effect on blood pressure, Varga et al. (1996) termed it triphasic. They argued that the prolonged hypotension is not centrally, but peripherally mediated by presynaptic inhibition of norepinephrine release from peripheral sympathetic nerve terminals (see Figure 3). Niederhoffer et al. (2003) confirmed the increase in vagal tone by cannabinoid agonists.

The first phase of the triple effect has been brought into context with the Bezold-Jarisch reflex: bradycardia, vasodilation, and hypotension as a result to stimulation of cardiac receptors or mechanical stimulation, mediated by vagal afferences (Aviado and Guevara Aviado, 2001; Warltier et al., 2003; Crystal and Salem, 2012). An activation of the reflex was shown to be induced by vanilloid receptor VR1 on the vagal nerve (Malinowska et al., 2001). Furthermore, Godlewski et al. (2003) showed non-CB receptor mediated modulation of the Bezold-Jarisch reflex by cannabinoid receptor agonists (e.g., WIN 55,212-2) via an inhibition of 5-HT_3_ receptors in cardiopulmonary afferent C-fibers. For a comprehensive overview on triphasic blood pressure responses *in vivo*, see the review by Malinowska et al. (2012).

#### Vasoconstriction and vasodilation in peripheral vasculature

The first indications of coronary artery constriction by Δ-THC came in 2005 from isolated heart experiments (Wagner et al., 2005). The vasoconstrictive action of Δ-9-THC could be shown for the first time on rat aortic rings by O'Sullivan et al. (2005b). Substance- and vessel-dependent vasoconstrictive reaction to Δ-THC and AEA in coronary and mesenteric vessels was demonstrated in the same year (O'Sullivan et al., 2005a; Wagner et al., 2005). The same group then demonstrated a slowly developing, time-dependent THC-induced vasodilation in aortic rings by an induction of PPARs, thus acting on the transcriptome of the targeted cells (O'Sullivan et al., 2005c, 2009). Vasorelaxation continued to increase over the course of 2 h in endothelium-intact specimen and was dependent on active nitric oxide and hydrogen peroxide production. As Kosersky (1978) and O'Sullivan et al. (2007) observed a vasorelaxant response in vessels of hypertensive rats (by chronic inhibition of nitric-oxide synthase). However, this effect was only seen *in vitro* and not *in vivo*, where the authors rather found a vasoconstrictor response.

#### CB-receptor independent effects

A CB-receptor independent vasodilative effect was described as a result to the action of AEA on voltage-gated calcium channels, the TRPV1, commonly referred to as endovanilloids (White and Hiley, 1998; Ho and Hiley, 2003). Cannabinoids have been proven to exert vasodilation independent of the endothelium or vanilloid- and cannabinoid-receptors (Breyne et al., 2006; Ho and Gardiner, 2009; Mair et al., 2010). THC is dependent on and interacting with H_2_O_2_ and acts differentially on different arterial types (O'Sullivan et al., 2006). In isolated perfused lungs, vasoconstriction as a result to AEA administration was independent of CB1 and CB2 receptors, but dependent on AEA metabolization by fatty acid amide hydrolase (FAAH) (Wenzel et al., 2013).

#### CB-receptor dependent effects

A receptor-dependent vasoconstrictor response has been reported for the CB1 receptor in spontaneously hypertensive rats (Wheal et al., 2007) and mesenteric arteries (Gardiner et al., 2009; Tamaki et al., 2012). Conversely, in rat aortic rings, the selective CB1 receptor agonist arachidonylcyclopropylamide (ACPA) caused vasodilation through activation of K_*Ca*_1.1 potassium- and inhibition of Ca_*V*_1.2 calcium-channels (Sánchez-Pastor et al., 2014). The CB1 receptor has otherwise been suspected to inhibit sympathetic neurogenic vasoconstrictor responses (Pakdeechote et al., 2007) and mostly shown vasodilative effects (Iring et al., 2013; Al Suleimani et al., 2015; Baranowska-Kuczko et al., 2016). Initial blood pressure and anesthesia influence the arterial response to cannabinoids with their subsequent haemodynamic profile, as demonstrated in conscious rats (Gardiner et al., 2009; Ho and Gardiner, 2009).

### Vascular effects of cannabinoids in larger mammals

#### Peripheral vasodilation and vasoconstriction in the lungs of dogs

Early *in vivo* experiments in anesthetized dogs by Cavero et al. (1972) revealed a reduction of cardiac output after i.v. administration of Δ-9-THC. A following publication additionally demonstrated the reactive reduction in heart rate and blood pressure (Cavero et al., 1973). Beaconsfield et al. (1972) had found an increase in cerebrovascular venous outflow in dogs after an i.v.-dose of as much as 100 μg/kg of Δ-9-THC. In contrast to this, Cavero et al. (1974) observed a reduction in venous return due to an action of Δ-9-THC in the splanchnic vasculature. The only vasoconstrictive effects in dogs were found in pulmonary vessels. Jandhyala et al. (1976) proposed a reflexogenic mechanism by which an increase in pulmonary blood pressure in dogs was induced by Δ-9-THC, independent of a secondary neurogenic reaction involving prostaglandins.

#### Vasopressor potentiation and peripheral vasodilation in cats

Graham and Li (1973) published analogous results to their observations in rats. Following the injection of cannabis extract and Δ1-THC, they potentiated a noradrenaline-induced systemic vasopressor response and delayed the direct vasoconstrictor response in hind legs of cats (Graham and Li, 1973). In an experiment on isolated cerebral arteries of the cat, vasodilation was identified specifically as a result of regulation of Ca^2+^-currents by increase of CB1 receptor activity through AEA (Gebremedhin et al., 1999). This showed the receptor's role in the increase of regional cerebral blood flow.

#### Vasodilation in cow and sheep

A vasodilative effect concomitant with an exposure to AEA's metabolite arachidonic acid has been shown in isolated bovine coronary artery rings (Pratt et al., 1998). The vasodilation was endothelium- but not CB-receptor-dependent and amounted to a maximum of 50%. A similar effect was observed in endothelium-intact ovine coronary rings. A maximum relaxation of about 80% was a supposed response to the presence of prostanoids converted from AEA by FAAH (also see Figure 2 and Grainger and Boachie-Ansah, 2001).

#### Vasoconstriction in rabbit ear arteries and cerebral and peripheral vasodilation

In the perfused rabbit ear artery, Δ9-THC induced vasoconstriction (Barbosa et al., 1981). Conversely, a vasodilative effect of Δ9-THC and AEA could be shown in isolated cerebral rabbit arteries, involving the metabolism of arachidonic acid and lower doses of the molecules (Ellis et al., 1995). Vasodilatation was induced in rabbit mesenteric arteries by AEA (cyclo-oxygenase-(COX)-dependent) and Δ9-THC (COX-independent) but not in carotid arteries of rabbits (Fleming et al., 1999). AEA and Δ9-THC appeared to inhibit the production of endothelial derived hyperpolarizing factor (EDHF) by an activation of the CB1 receptor. By injecting CB agonists directly into the cisterna cerebromedullaris in conscious rabbits, Niederhoffer and Szabo (2000) elicited an activation of the sympathetic nervous system as measured by an increase in renal sympathetic nerve activity and bradycardia. The increase of pulmonary arterial pressure in isolated lungs was likely mediated by COX-2-metabolites after AEA perfusion (Wahn et al., 2005).

#### Peripheral vasodilation and vasoconstriction in pigs, “human-like” metabolization of THC

In pig coronary arteries Fleming et al. (1999) observed no further vasodilation than that induced via the CB1 receptor by reduction of EDHF-synthesis second to Δ9-THC administration. The vasodilation was directly related to the inhibition of EDHF, confirming views of an active role of endocannabinoids in the regulation of vascular tone, dependent on the local endothelium (Brandes et al., 2002). In search of a novel model system for cannabinoid metabolism, Brunet et al. (2006) demonstrated the validity of the pig as a suitable organism to study Δ9-THC kinetics *in vivo*. They found intense hepatic metabolism and a high concentration of Δ9-THC in lungs and fatty tissues like the brain (Brunet et al., 2006). This distribution was confirmed in a post-mortem study after i.v.-injection of Δ9-THC (Brunet et al., 2010). The selective constrictive vascular effect of Δ9-THC has been postulated in liver microvasculature in a pharmacological study in combination with Celecoxib in a porcine model of hemorrhagic shock (Vaddady et al., 2011). Celecoxib is a selective inhibitor of COX 2, which converts arachidonic acid to endogenous vasodilator and platelet inhibitor precursor prostaglandin H2 (also see Figure 2). In Abn-CBD perfused retinal arterioles, vasorelaxation was mediated in precontracted retinal vessels (Su et al., 2015).

### Results summary

#### Cannabinoids' effects in cerebral vasculature

In total, we identified nine studies addressing cannabinoids' effects on cerebral vasculature (for an overview see Table 1). Of these, four involved *in vivo* experiments on rodents, in which CBF was measured as a read-out for variation in cerebrovascular tone. CBF variation in these studies was probably secondary to neuronal activation or deactivation rather than the translation of a direct vascular effect (Bloom et al., 1997; Stein et al., 1998; Zhang et al., 2008; Iring et al., 2013). In two of these four experiments, the animals remained conscious for the time of drug administration and measurements (Bloom et al., 1997; Stein et al., 1998). Direct measurements on isolated arteries in three other publications showed cerebrovascular dilation in reaction to cannabinoid perfusion (Hillard et al., 2007; MacIntyre et al., 2014; Su et al., 2015). Vasodilation decreases peripheral resistance and increases blood flow. In the cerebral territory, this may be a protective mechanism and increase oxygen supply in case of a cerebral insult. Depending on the time-point at which vasodilation is activated, this protection may be beneficial in early stages of ischemia. If it occurs at a later stage, it might accelerate the recuperation of cerebral function.

**Table 1:** Cannabinoid's vascular effects in animal models specifically targeting cerebral vessels.

**Animal model**	**Reference**	***in vitro in vivo***	**Consc**.	**Bed**	**Molecule**	**Dose**	**Appl**.	**Targ**.	**Effect**
Rat	Bloom et al., 1997	*in vivo*	consc.	Cerebral	Δ9-THC	0.5 mg/kg 1 mg/kg 4 mg/kg 16 mg/kg	i.v.	unsp.	increase and decrease of CBF depending on region (see text for details)
					11-OH-THC	4 mg/kg	i.v.	unsp.	Increase and decrease of CBF
Rat	Stein et al., 1998	*in vivo*	consc.	Cerebral	AEA	3mg/kg	i.v.	CB1r	no effect
						10mg/kg	i.v.	CB1r	rCBF↓ in 7 areas (including amygdala, cingulate, frontal, prepyriform, sensorimotor, claustrocortex)
						30mg/kg	i.v.	CB1r	rCBF↓ in 23 areas (including CA1 and CA3 of hippocampus, rostral core portion of nucleus accumbens, rostral caudate nucleus)
Rat	Hillard et al., 2007	*in vitro*	−	Isolated MCA	2-AG	5-10,000nM	perf.	CB1r	attenuation of U-46619 induced vasoconstriction
Rat	Iring et al., 2013	*in vivo*	ur. g.a	Cerebral	AM-251	10 mg/kg	i.v.	unsp.	no effect on CoBF
					AM-404	10 mg/kg	i.v.	unsp.	initial CoBF↑, systemic BP↑, followed by CoBF↓ and BP↓
Rat	MacIntyre et al., 2014	*in vitro*	−	Isolated retinal arterioles	Abn-CBD	perf.	10 μM	GPR, CB_*E*_	inhibition of endothelin-induced vasoconstriction, endothelium-dependent, involving SK_*Ca*_ channels
Mouse	Zhang et al., 2008	*in vivo*	ket-xy g.a.	Cerebral	O-1966	1 mg/kg	i.v. i.p.	CB2r	rCBF↑
Rabbit	Ellis et al. (1995)	*in vitro*	−	Cerebral	Δ9-THC	10^−13^-10^−3^M	perf.	unsp.	Dose-dependent vasodilation
					AEA	10^−13^-10^−3^M	perf.	CB1r	Dose-dependent vasodilation
Cat	Gebremedhin et al. (1999)	*in vitro*	−	Isolated cerebral arteries	R-(+)-WIN 55, 212-2	10-100nM	perf.	CB1r	L-type Ca^2+^-current↓
					AEA	10-300nM	perf.	CB1r	L-type Ca^2+^-current↓
Pig	Su et al. (2015)	*in vitro*	−	Retinal arterioles	Abn-CBD	10^−10^to 10^−4^M	perf.	CB_*E*_	vasorelaxation in pre-contracted vessels, action on endothelium

*Consc, consciousness; Appl, application mode; Targ, target; consc, conscious; THC tetrahydro-cannabinol; i.v, intravenous; unsp, unspecified; CBF, cerebral blood flow; AEA, anandamide; CB1r, cannabinoid receptor type 1; MCA, middle cerebral artery; U-46619, thromboxane A_2_; Abn-CBD, abnormal cannabidiol; GPR, G-protein coupled receptor 18; CB_E_, novel endothelial receptor; CB2r, cannabinoid receptor type 2; rCBF, regional cerebral blood flow; ur, urethane; g.a, general anaesthesia; CoBF, cerebrocortical blood flow; BP, blood pressure; ket-xy, ketamine-xylazine; i.p, intraperitoneal; perf, perfusion*.

The remaining two studies utilized isolated cerebral vessels of rabbits (Ellis et al., 1995) and cats (Gebremedhin et al., 1999). In rabbit and cat brain arterioles, the CB1 receptor mediates vasodilation, probably following an inhibition of Ca^2+^-influx in cerebral arterial muscle cells and possibly involving arachidonic acid metabolism. While nine different cannabinoids were employed overall, there were three studies that at least partially indicated vascular tone increase (i.e., contraction) as a reaction to the drugs (Bloom et al., 1997; Stein et al., 1998; Iring et al., 2013). All of the latter were conducted on rats.

#### Cannabinoids' effects in peripheral vasculature

Cannabinoids exert a vasodilative and cardio-depressor effect with a concomitant reduction in heartrate, cardiac output, and blood pressure as well as an increase in cerebral blood flow in dogs. AEA caused vasodilation in both bovine and ovine coronary artery rings. The endothelium mediated this effect by metabolization via FAAH. In the rabbit, there was evidence for a vasoconstrictive effect of Δ9-THC in ear arteries. In pulmonary arteries an increase of arterial pressure following AEA perfusion could be observed. In other vascular beds, cannabinoids and CB1 receptor activation resulted in vasodilation, partly involving cyclo-oxygenase activation. In the pig, coronary arteries did not react to cannabinoid-perfusion. There is evidence that Δ9-THC may have vasoconstrictive effects in selected vascular beds. Tables S1, S2 in the supplementary material section give a detailed overview of all cited publications and the corresponding respective vasodilative (S1) or vasoconstrictive effects (S2), ordered by animal species.

## Discussion

Animal experiments show complex circulatory and vascular responses as a result to the administration of cannabinoids, dependent on a variety of factors such as the specific molecules, their doses, the animal model, the experimental preparation, and the particular vascular bed. In this review, the presented studies confirm that administration of cannabinoids can lead to vasodilation in a number of animal models. Our literature review also provides evidence for vasoconstrictive effects of cannabinoid administration. While vasodilation may improve blood perfusion and protect against ischemia, severe vasoconstriction can result in hypoperfusion by a reduction of CBF. In the cerebral vascular bed, vasoconstriction could corroborate clinical observations hinting to an etiologic involvement of cannabis consumption in acute cerebral stroke incidence of young patients. Animal studies targeting cannabinoids' effects on the the cerebral vascular bed are scarce.

### The role of the endocannabinoid system and cannabis consumption in vascular reactivity and in stroke

The past 15 years have given rise to the discussion of neuroprotective and beneficial effects of cannabinoids and the endocannabinoid system in stroke (England et al., 2015), for example by a reduction of infarct volume (Mishima et al., 2005). The endocannabinoid system does not seem to be altering the incidence of cerebral ischemia. It exerts a protective effect via an activation of the CB1 and CB2 receptors. The latter play a differential role in the development of atherosclerosis (Mach et al., 2008) and improve post-stroke recuperation by neuroblast activation (Bravo-Ferrer et al., 2017). The modulation of the endocannabinoid system and notably of the CB2 receptor response has recently been shown in alcohol-induced dilation of fetal cerebral arteries in a primate model (Seleverstov et al., 2017). CB2 receptors may play a more important role in cerebral vascular regulation than has been postulated before. Vasodilation could provide a protective effect by maintaining CBF in early stages of ischemia. The recent review by Benyó et al. (2016) gives an extensive overview of the complex interactions of endocannabinoids and CB receptors in cerebrovascular regulation. The authors point to the fact that the protective mechanisms mediated by the CB2 receptor may come into play in the subacute phases after the primary insult.

In humans, the consumption of cannabinoids, particularly by combustion and inhalation, has been associated with the occurence of cerebral infarcts (Garrett et al., 1977; Wolff et al., 2015c; Rumalla et al., 2016b; Wolff and Jouanjus, 2017). Nevertheless, the underlying pathophysiological characteristics are poorly understood. Cannabis and synthetic cannabinoid consumption may be a potential trigger factor for reversible intracranial vasoconstriction, a pathology that manifests with cerebral infarcts in up to 39% (Wolff et al., 2015a). Vasoconstriction with severe reduction of CBF in the dependent territories could be a mechanism causing neuronal death through ischemia. The high frequency of posterior circulation stroke due to cannabis consumption (Wolff et al., 2014; Wolff and Jouanjus, 2017) could prove the study of vertebro-basilar territories to be particularly interesting. Cellular damage by oxidative stress and mitochondrial dysfunction following *in vitro* administration of THC has been discussed (Wolff et al., 2014, 2015b). The possible link of mitochondrial dysfunction to vasoconstriction remains to be clarified. A selective inhibition of cerebral mitochondrial monoamine oxidase (MAO) by Δ1-THC and cannabidiol when compared to liver mitochondria in the pig has been evoked, showing organ specific succeptibility (Schurr and Livne, 1976; Schurr et al., 1978). More recently, a receptor and non-receptor mode of action of cannabinoids to inhibit pig mitochondrial respiratory function has been proposed (Fišar et al., 2014). To elucidate mechanisms that could explain how cannabinoids are involved in the pathophysiology of brain function and stroke, especially in young patients, the substance's study in animal models is warranted and seems requisite. As cannabinoids are perceived to mostly exert vasodilation in peripheral vessels, it seems necessary to further investigate vasoconstrictive action of these compounds, particularly in respect to cerebral vessels and a possible implication in stroke etiology. Clinical evidence has been accumulating, as stated above, to support this need. Vasoconstriction may well represent a mechanism by which cannabinoids could be acutely harmful and a potential cause for cerebral ischemia.

### Animal studies on cannabinoids' effects in cerebral vasculature

The results of nine studies focusing on cerebral vascular territories were heterogenous concerning cannabinoids' effects on vascular tone. In rodents both vasodilation and vasoconstriction could be observed after Δ9-THC, 11-OH-THC, AEA, and AM-404 exposure. Abn-CBD and O-1966, a CB2r agonist, rather provoked vasodilation. In larger mammals, all perfused cerebral vessels reacted with wall-relaxation. Due to the high variety of animal models, experimental setup, cannabinoid molecules, and their doses, it seems difficult to draw any generalizable conclusions from these data. At the same time, there is clear indication of concomitant vasoconstriction to cannabinoid exposure. In the past, cannbinoid exposure has predominantly been brought into context with vasodilation.

### Peripheral vasodilation and vasoconstriction induced by cannabinoids

We found numerous studies in which a vasodilative effect of cannabinoids was shown in the peripheral vasculature of rodents. Most of these experiments have found a systemic response with blood pressure depression, which was partly mediated by CB receptor action.

Early experimental results in the rat have hinted to a vasoconstrictive activity of THC comparable to that of norepinephrine (Adams et al., 1976). The fact that a simple vasodilation does not account for all of the substances' complex influences on cardiovascular tone was strengthened by the description of the “triphasic” or “triple” effect (Siqueira et al., 1979; Varga et al., 1996; Malinowska et al., 2012). Interestingly, a hypertensive reaction was described in dose-dependent manner in freely moving animals in the first study that did not employ general anesthesia (Kawasaki et al., 1980). The hypertensive effect of stress conditions, such as conscious restraint, hypoxia, or hypercapnia in conjunction with endocannabinoids has been highlighted in the review by Benyó et al. (2016). Most of the reviewed experiments were conducted under general anesthesia and with i.v.-injection of the respective cannabinoids. A total of 19 of the 51 included papers stated constrictive effects of cannabinoids in blood vessels (see Table S2 in Supplementary Material for overview).

More recent studies show CB receptor-dependency and the influence of factors such as time, anesthesia, prior baseline arterial pressure, vessel origin, or location and substance specifics on the vascular effect of the compounds. The dose of specific compounds can have a direct influence on the presence and intensity of its effects (Kawasaki et al., 1980; Stein et al., 1998; Mair et al., 2010). An alteration of the dose can also inverse the effect from vasodilation at low concentrations to vasoconstriction at higher concentrations in the case of AEA (Tamaki et al., 2012). Moreover, metabolization is a determining factor in the activation of AEA-induced vasoconstriction in pulmonary arteries. Vasoconstriction was dependent on AEA-hydrolysis to arachidonic acid by FAAH (Wenzel et al., 2013). In addition, THC acts differentially on different arterial types (O'Sullivan et al., 2006) and the action of THC can be dependent not only on the arterial type but also on the choice of either an *in vivo* or an *in vitro* model (O'Sullivan et al., 2007). This shows how important the selection of the model system is, if an approach to answer clinical questions is to be made.

### Animal models in cardiovascular research on cannabinoids—a possible outlook

The importance of the choice of a model system was underlined by O'Sullivan et al. in the recent review and meta-analysis by Sultan et al. (2018). They systematically searched for publications on the haemodynamic effects of THC in humans and *in vivo* animal models and extracted data on the doses, changes in blood pressure, heart rate, and blood flow, experimental conditions and each model used. In humans heart rate was monitored. Overall, blood pressure, heart rate, and blood flow changes were heterogenous and depended on the species, consciousness level, anaesthetic agent, and administration mode. This seems to be in line with our observations regarding cannbinoids' effects on vasculature *in vivo* and *in vitro*. The authors underline the need for further investigations of THC's haemodynamic effects in humans. Studying THC's effects on circulation in humans may prove ethically challenging. Accordingly, the search for the most adequate animal model has not ceased.

The quality of an animal model for toxicological studies can be influenced by different factors of anatomical nature. On a functional level, comparability of vasculature influences a models' relevance for the human anatomy and physiology. Substances like THC, that are subject to intra-corporal metabolization, can exert differing effects as their respective metabolites differ. Δ9-THC metabolism has been studied in rats, where a low retention was observed in the brain. Interestingly, brain retention was higher after administration by inhalation, than by i.v.-administration (Leighty, 1973). This may be in line with clinical observations of cardiovascular events after inhalation of high doses of marijuana. Harvey (1988) have analyzed the *in vivo* metabolism in mice exclusively after intraperitoneal injection and in comparative *in vitro* studies in seven species, including rabbits and cats, showing considerable dependency on species for metabolic hydroxylation sites (Harvey and Brown, 1990, 1991). Brunet et al. (2010) argue that these smaller species require much higher doses in drug administration to reach comparable tissue concentration levels when compared to humans, as shown in tissue distribution studies.

To us, the need for an appropriate animal model for cerebral *in vivo*-studies on cannabinoids' vascular effects seems evident. We esteem that the pig may present such a model. Pigs have been attractive to researchers examining pharmaceuticals because of the possibility of recurrent and extensive tissue and blood sampling as well as their physiological characteristics that resemble the human species more closely than alternatives. Brunet et al. (2006) were able to show that cannabinoid pharmacokinetics and retention in fatty tissue (like brain white matter) and fat of the large white pig were comparable to observations that have been made in heavy cannabis users. They proposed a wider application of the model for cannabinoid metabolism. “Human-like” sensitivity to THC and comparable metabolization via 11-OH-THC are two more advantages of this model (Brunet et al., 2006; Huestis, 2007; Schaefer et al., 2016). Neuroanatomical studies and functional studies of the cortex have shown a considerable similitude to humans in embryologic development and postnatal development of the brain (Sauleau et al., 2009). Pig brains are considerably larger than those of rodents and therefore accessible to elaborate imaging techniques, such as angiographies. They exibit a posterior cerebral circulation as well as a circle of Willis (Jablonski et al., 1989; Oliveira and Campos, 2005). The vascular tone of both porcine and bovine basilar arteries can be modulated by COX-inhibition (decrease) and by nitric oxide inhibition (increase), but bradykinin only evokes basilar artery relaxation in pigs Miyamoto et al. (1994). This finding again stresses the importance of the choice of the most adequate model species. The porcine basilar artery has repeatedly served as a model for vascular tone studies (Miyamoto et al., 1998, 2006).

The focus of the past forty years of research in this field has mostly been set on rodent models, supposedly for reasons of feasibility and cost. Nonetheless, their low sensitivity to THC distorts experimental conditions, compared to the human. Most other large mammals are comparably expensive. Pigs are in general easily accessible, due to commercial consumption. The pig may present a valid animal model for brain disorders and for the study of pharmacokinetics of cannabinoids (Lind et al., 2007; Schaefer et al., 2015, 2016). Fast growth and early sexual maturity in pigs would favor age related studies. This may be of particular interest, given the observations of stroke and cannabis consumption in young patients.

### Limitations and strengths

Our systematic literature review was limited by the low number of publications targeting the cerebral vasculature. We identified nine studies on cerebral vessels according to our inclusion criteria (see Table 1). A less restrictive association of keywords in our search algorithm, might have widened our results. We did, however, employ general search terms and an association with clinical keywords, in order to approach our hypothesis of an association of stroke incidence with cannabis consumption. The heterogeneity of the animal species, experimental conditions and methodology require a classification to provide a comprehensive overview of the results. In some of the cited publications, baseline characteristics (such as blood pressure) were deliberately modified. Others avoided unphysiological alterations, even anaesthesia, as much as possible. Wherever applicable, we signaled these specifics.

We classified our search results by our particular initial field of interest, cerebral vasculature. We chronologically ordered identified studies by animal species to facilitate an overview of formerly employed models. While vasodilation can exert protective effects, vasoconstriction may be a detrimental mechanism linking cannabis consumption to ischemic stroke in humans. In order to provide an overview for the conception of future experiments examining the possible implication of cannabis in the pathophysiology of stroke, we present the identified studies by a classification of vasoconstriction versus vasodilation in the supplementary materials section (Tables S1, S2).

## Conclusion

Scientific data on the effect of cannabinoids in cerebral vascular regulation in animal models is slim. Evidence has been accumulating with both vasodilation and constriction occurring secondary to cannabinoid exposure and metabolization. Clinical data suggest that cannabinoid uptake may play a role in the etiology of cerebral infarcts, but the underlying mechanisms are unclear. Vasoconstriction may be the common macroscopic endpoint of a multitude of molecular mechanisms triggered by cannabinoids. Further research, possibly employing the pig as a model system, could accelerate our understanding of the role of cannabinoids in the incidence of cerebral stroke and the corresponding pathophysiology. It seems licit to consider these results and their potentially dangerous consequences in the ongoing discussions on marijuana legalization. Constituents of cannabis undoubtedly have complex regulatory effects on peripheral and cerebral vasculature. Psychotropic and medical benefits of cannabis in chronic pain, spasticity, and sedation should not be evaluated independently of these side effects.

## Author contributions

JRi, BG, and VW contributed conception and design of the review as well as the interpretation of the data. JRi wrote the first draft of the manuscript. VQ, OR, JRa, and RB made substantial contributions to the interpretation of the data and revised the work critically for intellectual content. All authors contributed to the manuscript revision, read and approved the submitted version.

### Conflict of interest statement

The authors declare that the research was conducted in the absence of any commercial or financial relationships that could be construed as a potential conflict of interest.
