# Supplementary material for: A Systematic Review of the Complex Effects of Cannabinoids on Cerebral and Peripheral Circulation in Animal Models
Source: Front Physiol. 2018 May 29;9:622. doi: 10.3389/fphys.2018.00622 (PMC5986896; doi:10.3389/fphys.2018.00622)
Supplement: Supplementary file 1 [file Image_1.pdf]

Supplementary Material: A systematic review of the complex effects of cannabinoids on cerebral and peripheral circulation in animal models

1 SUPPLEMENTARY TABLES AND FIGURES

1.1 Figures

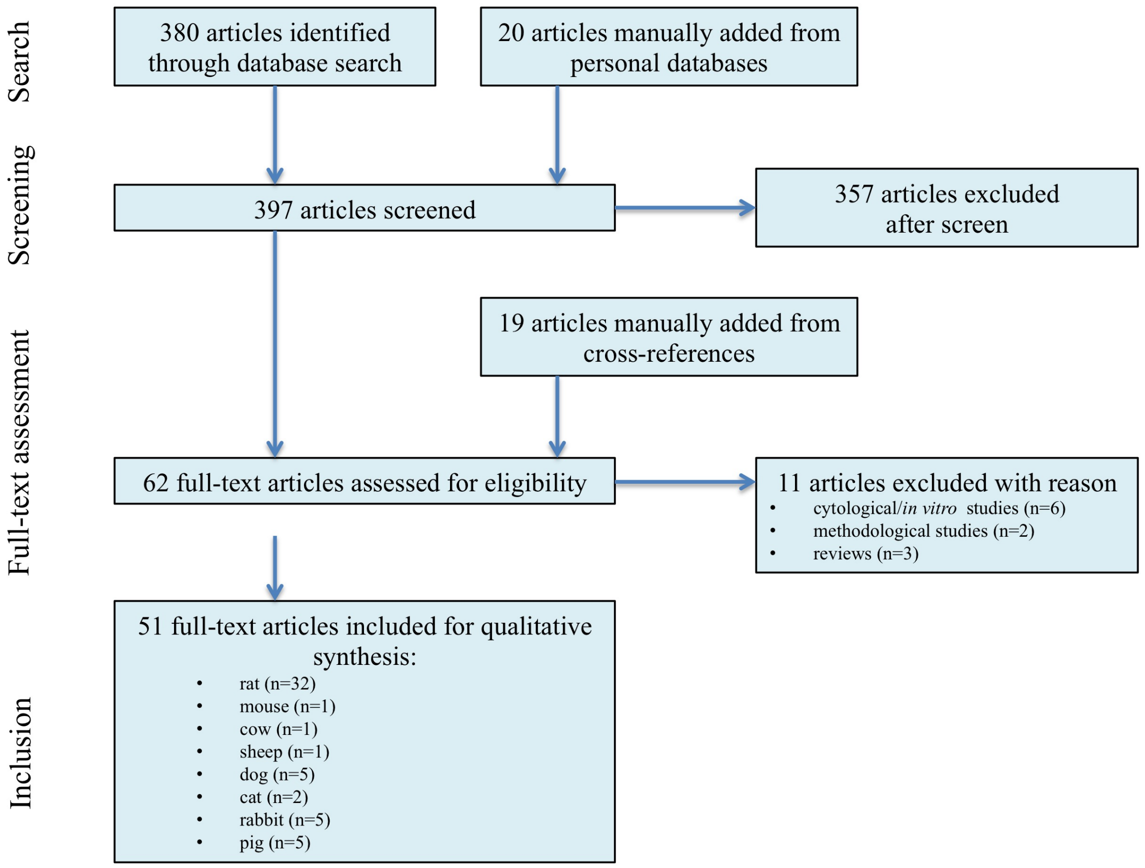

Figure S1. Article search and inclusion flow-chart

1.2 Tables

Table S1: Studies indicative of vasodilative effects of cannabinoids in animal models.

Animal model	Reference	*in vitro* *in vivo*	Consc.	Bed	Molecule	Dose	Appl.	Targ.	Effect
rat	Graham and Li (1973)	*in vivo*	ur. g.a.	hindqu.	c.e.	10mg/kg	i.v.	unsp.	systemic BP↓, HR↓, resp.rate↓
						50mg/kg/day 14 days	i.p.	unsp.	develops tolerance
					Δ1-THC	0.5mg/kg	i.v.	unsp.	as for c.e.
					Δ6-THC	0.5mg/kg	i.v.	unsp.	as for c.e.
					cannabinol	1mg/kg	i.v.	unsp.	no effect
					cannabidiol	1mg/kg	i.v.	unsp.	no effect
rat	Adams et al. (1976)	*in vivo*	ur. g.a.	systemic	Δ8-/Δ9-THC	unavailable	i.v.	unsp.	transient BP↑, followed by prolonged BP↓, HR↓
				hindqu.	Δ8-/Δ9-THC	unavailable	i.a.	unsp.	vasoconstriction
rat	Kosersky (1978)	*in vivo*	consc.	systemic	Δ9-THC	25mg/kg	oral	unsp.	systolic BP↓ in spontaneously hypertensive rats
rat	Siqueira et al. (1979)	*in vivo*	ur. g.a.	systemic	Δ9-THC	2-10mg/kg	i.v.	unsp.	immediate BP↓, HR↓, BP↑ at 30", persistent BP↓ at 5' (vagal?)
rat	Varga et al. (1996)	*in vivo*	ur. g.a.	systemic	AEA	4mg/kg	i.v.	CB1r	transient BP↓, brief BP↑, prolonged BP↓
rat	Bloom et al. (1997)	*in vivo*	consc.	cerebral	Δ9-THC	0.5mg/kg 1mg/kg 4mg/kg 16mg/kg	i.v.	unsp.	increase and decrease of CBF depending on region (see text for details)
					11-OH-THC	4mg/kg	i.v.	unsp.	increase and decrease of CBF
rat	Stein et al. (1998)	*in vivo*	consc.	cerebral	AEA	3mg/kg	i.v.	CB1r	no effect on rCBF

Table S1 continued

						10mg/kg	i.v.		rCBF↓ in 7 areas (including amygdala, cingulate, frontal, prepyriform, sensorimotor, claustrocortex)
						30mg/kg	i.v.		rCBF↓ in 23 areas (including CA1 and CA3 of hippocampus, rostral core portion of nucleus accumbens, rostral caudate nucleus)
rat	White and Hiley (1998)	*in vitro*	-	mes. art.	AEA	unsp.	perf.	CB1r	relaxation in all (precontracted)
					CP 55,940			CBr	
					HU-210			CBr	
					palmytoyl-ethanolamide			CB2r	
					R-(+)-WIN 55,212-2			CB2r	
rat	Ho and Hiley (2003)	*in vitro*	-	mes. art.	AEA	1μM	perf.	CB1r	relaxation in all (precontracted)
					JWH 015	30μM		CB2r	
					R-(+)-WIN 55,212-2	60μM		CB2r	
					S-(+)-WIN 55,212-3	60μM		unsp.	
rat	Niederhoffer et al. (2003)	*in vivo*	ur. g.a.	systemic	WIN55212-2	30-1000μ/kg	i.v.	unsp.	MAP↓, HR↓, plasma NA conc.↓ dose-dependently
					CP55940	30-1000μ/kg	i.v.	unsp.	MAP↓, HR↓, plasma NA conc.↓, max. at 30μg/kg
rat	Wagner et al. (2005)	*in vitro*	-	isolated heart	AEA	1-100nmol	bolus	CB1r	coronary vasodilation

Table S1 continued

					Delta-THC	10-100nmol	bolus	unsp.	coronary vasoconstriction
rat	O'Sullivan et al. (2005b)	*in vitro*	-	aortic rings	Δ9-THC	cumulative μM range	perf.	unsp.	vasorelaxation/-constriction
					AEA			CB1r	vasorelaxation
					NADA			CB1r	vasorelaxation
rat	O'Sullivan et al. (2005a)	*in vitro*	-	aortic and mes. rings	Δ9-THC	cumulative μM range	perf.	unsp.	vasorelaxation in smaller branches, Ca^{2+}-influx block; vasoconstriction in sup. mes. art. (stem) at high concentration; antagonism to AEA-vasorelaxation
rat	O'Sullivan et al. (2005c)	*in vitro*	-	aortic and mes. rings	Δ9-THC	10μM	perf.	unsp.	time-dependent vasorelaxation mediated by PPARγ
rat	O'Sullivan et al. (2006)	*in vitro*	-	isolated aorta	Δ9-THC	10μM, 2h incubation	-	unsp.	no effect
				mes. art.	Δ9-THC		-		enhanced endothelium-dependent vasorelaxation in sup. mes. art. and potentiated contraction in resistance vessels
rat	Breyne et al. (2006)	*in vitro*	-	gastric artery	methan-andamide	100μmol/L	perf.	CB1r	dose-dependent relaxation, probable Ca^{2+}-influx↓
rat	O'Sullivan et al. (2007)	*in vitro*	consc.	isolated mes. art.	Δ9-THC	cumulative μM range	perf.	unsp.	enhanced vasorelaxation in hypertensive rats
		in vivo		hindqu. and systemic	Δ9-THC	1mg	i.v.	CB1r	mediated vasopressor effect (long-lasting BP↑), not enhanced by hypertension

Table S1 continued

rat	Wheal et al. (2007)	*in vivo*	consc.	systemic	AEA	3mg/kg	i.v.	CB1r	hypotension in spontaneously hypertensive rats
					R-(+)-WIN 55,212-2	150μg/kg	i.v.	CB1r	hypotension in spontaneously hypertensive rats
rat	Pakdeechote et al. (2007)	*in vitro*	-	isolated mes. art.	AEA	1μM	perf.	CB1r	CB1r-mediated attenuation of neurogenic vasoconstriction
					R-(+)-WIN 55,212-2	1μM	perf.	CB1r	non CB1r-mediated attenuation of neurogenic vasoconstriction
					CP55940	1μM	perf.	CB1r	non CB1r-mediated attenuation of neurogenic vasoconstriction
rat	Hillard et al. (2007)	*in vitro*	-	isolated MCA	2-AG	5-10000nM	perf.	CB1r	attenuation of U-46619 induced vasoconstriction
rat	O'Sullivan et al. (2009)	*in vitro*	-	aorta	cannabidiol	1μM	perf.	unsp.	time-dependent vasorelaxation, in part mediated by PPARγ
rat	Ho and Gardiner (2009)	*in vivo*	consc.	systemic	AEA	3mg/kg	i.v.	CB1r	dose-dependent MAP$\downarrow$ in hypertensive rats, no effect in normotensive rats
					R-(+)-WIN 55,212-2	150μg/kg	i.v.	CB1r	MAP$\downarrow$ in hypertensive rats, MAP$\uparrow$ in normotensive rats
rat	Gardiner et al. (2009)	*in vivo*	consc.	systemic	methan-andamide	0.5-5mg/kg	i.v.	CB1r	CB1r-mediated renal and mesenteric vasoconstriction and hindquarter vasodilation
rat	Mair et al. (2010)	*in vitro*	-	coronary artery	AEA	1nM-30μM	perf.	CB1r	concentration-dependent vasorelaxation via a sphingosine kinase-1 mechanism
rat	Tamaki et al. (2012)	*in vitro*	-	mes. art.	AEA	0.1-1nM	perf.	CB1r	vasodilation
						10nM-1μM	perf.	CB1r	vasoconstriction

Table S1 continued

rat	Iring et al. (2013)	*in vivo*	ur. g.a	cerebral	AM-251	10mg/kg	i.v.	unsp.	no effect on CoBF
					AM-404	10mg/kg	i.v.	unsp.	initial CoBF↑, systemic BP↑, followed by CoBF↓ and BP↓
rat	Sánchez-Pastor et al. (2014)	*in vitro*	-	aortic rings	ACPA	0.1-50μM	perf.	CB1r	vasorelaxation involving activation of K_{Ca}1.1- and inhibition of Ca_V1.2-channels
rat	MacIntyre et al. (2014)	*in vitro*	-	isolated retinal arterioles	Abn-CBD	10μM	GPR18perf. CB$_E$		inhibition of endothelin-induced vasoconstriction, endothelium-dependent, involving SK_{Ca} channels
rat	Al Suleimani et al. (2015)	*in vitro*	-	sup. mes. art.	O-1601	10nM-100μM	perf.	CB1r	vasorelaxation partly endothelium-mediated in third order branch
rat	Baranowska-Kuczko et al. (2016)	*in vitro*	-	aortic and mes. art. rings	methan-andamide	0.01-30μM	perf.	CB1r	vasorelaxation of pre-constricted vessels
mouse	Zhang et al. (2008)	*in vivo*	ket-xy g.a.	cerebral	O-1966	1mg/kg	i.v. i.p.	CB2r	rCBF↑
rabbit	Ellis et al. (1995)	*in vitro*	-	cerebral	Δ9-THC	$10^{-13}$to 10^{-3}M	perf.	unsp.	dose-dependent vasodilation
rabbit	Fleming et al. (1999)	*in vitro*	-	mes. art. rings	HU 210	μM range	perf.	CB1r	relaxation
				Δ9-THC	μM range	perf.	CB1r	CB1r	relaxation
					AEA	0.03-30μM	perf.	CB1r	slowly developing relaxation involvement of EDHF, activated by CB1r

Table S1 continued

				carotid art. rings	HU 210	μM range	perf.	CB1r	no effect
					Δ9-THC	μM range	perf.	CB1r	no effect
					AEA	0.03-30μM	perf.	CB1r	no effect
dog	Cavero et al. (1972)	*in vivo*	s-p g.a.	systemic	Δ9-THC	2.5mg/kg	i.v.	unsp.	BP↓, HR↓, cardiac output↓, splanchnic vasoconstriction
dog	Cavero et al. (1973)	*in vivo*	s-p g.a.	systemic	Δ9-THC	39μg/kg 312.5μg/kg 2.5mg/kg	i.v.	unsp.	aortic BP↓, cardiac output↓, left ventricular peak pressure↓, left ventricular and diastolic pressure↓, no effect on cerebral stroke volume
dog	Cavero et al. (1974)	*in vivo*	s-p g.a.	systemic	Δ9-THC	2.5mg/kg	i.v.	unsp.	dose-dependent BP↓, HR↓, cardiac output↓, right ventricular contractile force↓, total peripheral vascular resistance↓
cat	Graham and Li (1973)	*in vivo*	ur. g.a.	hindqu.	c.e.	10mg/kg	i.v.	unsp.	systemic BP↓
					Δ1-THC	0.2mg/kg	i.v.	unsp.	pulse↓, resp. rate↓ hindlimb PP↓
					AEA	$10^{-13}$to 10^{-3}M	perf.	CB1r	dose-dependent vasodilation
cat	Gebremedhin et al. (1999)	*in vitro*	-	isolated cerebral arteries	R-(+)-WIN 55,212-2	10 to100nM	perf.	CB1r	L-type Ca^{2+}-current↓
					AEA	10 to 300nM	perf.	CB1r	L-type Ca^{2+}-current↓
cow	Pratt et al. (1998)	*in vitro*	-	coronary art. rings	AEA	cumulative μM range	perf.	CB1r	non CB1r-mediated vasodilation

Table S1 continued

sheep	Grainger and Boachie-Ansah (2001)	*in vitro*	-	coronary art. rings	AEA	0.01-30μM	perf.	CB1r	concentration-dependent relaxation, in part endothelium-dependent, in part K^+-channel mediated
pig	Su et al. (2015)	*in vitro*	-	retinal arterioles	Abn-CBD	$10^{-10}$to 10^{-4}M	perf.	GPR, CB_E	vasorelaxation in pre-contracted vessels, action on endothelium

Abbreviations: Consc. consciousness, Appl. application mode, Targ. target, ur. urethane, g.a. general anesthesia, hindqu. hindquarters, c.e. cannabis extract, i.v. intravenous, unsp. unspecified, BP blood pressure, HR heart rate, resp. respiratory , THC tetrahydrocannabinol, i.a. intraarterial, i.p. intraperitoneal, consc. conscious, AEA anandamide, CB1r cannabinoid receptor type 1, CB2r cannabinoid receptor type 2, CBF cerebral blood flow, rCBF regional cerebral blood flow, mes. mesenteric, art. artery, perf. perfusion, MAP mean arterial pressure, NA conc. noradrenaline concentration, max. maximum, CoBF cerebro-cortical blood flow, NADA N-arachnidonoyl-dopamine, MCA middle cerebral artery, Abn-CBD abnormal cannabidiol sup. superior, PPARγ Peroxisome proliferator-activated receptor gamma, ACPA arachidonylcyclopropylamide,ket-xy ketamine-xylazine, EDHF endothelium-derived hyperpolarizing factor, s-p sodium-pentobarbital, PP perfusion pressure, abnCBD abnormal cannabidiol, GPR G-protein coupled receptor 18, CB_E noncannabinoid endothelial receptor.

Table S2: Studies indicative of vasoconstrictive effects of cannabinoids in animal models.

Animal model	Reference	*in vitro* *in vivo*	Consc.	Bed	Molecule	Dose	Appl.	Targ.	Effect
rat	Adams et al. (1976)	*in vivo*	ur. g.a.	systemic	Δ8-/Δ9-THC	unavailable	i.v.	unsp.	transient BP↑, followed by prolonged BP↓, HR↓
				hindqu.	Δ8-/Δ9-THC	unavailable	i.a.	unsp.	vasoconstriction
rat	Siqueira et al. (1979)	*in vivo*	ur. g.a.	systemic	Δ9-THC	2-10mg/kg	i.v.	unsp.	immediate BP↓, HR↓, BP↑ at 30", persistent BP↓ at 5' (vagal)
rat	Kawasaki et al. (1980)	*in vivo*	ur. g.a.	systemic	Δ9-THC	1mg/kg 2mg/kg 5mg/kg	i.v.	unsp.	transient dose-related BP↑, HR↓ (vagal)
rat	Varga et al. (1996)	*in vivo*	ur. g.a.	systemic	AEA	4mg/kg	i.v.	CB1r	transient BP↓, brief BP↑, prolonged BP↓
rat	Bloom et al. (1997)	*in vivo*	consc.	cerebral	Δ9-THC	0.5mg/kg 1mg/kg 4mg/kg 16mg/kg	i.v.	unsp.	increase and decrease of CBF depending on region (see text for details)
					11-OH-THC	4mg/kg	i.v.	unsp.	increase and decrease of CBF
rat	Stein et al. (1998)	*in vivo*	consc.	cerebral	AEA	3mg/kg	i.v.	CB1r	no effect on rCBF
						10mg/kg	i.v.		rCBF↓ in 7 areas (including amygdala, cingulate, frontal, prepyriform, sensorimotor, claustrocortex)
						30mg/kg	i.v.		rCBF↓ in 23 areas (including CA1 and CA3 of hippocampus, rostral core portion of nucleus accumbens, rostral caudate nucleus)

Table S2 continued

rat	Wagner et al. (2005)	*in vitro*	-	isolated heart	AEA	1-100nmol	bolus	CB1r	coronary vasodilation
					Delta-THC	10-100nmol	bolus	unsp.	coronary vasoconstriction
rat	O'Sullivan et al. (2005b)	*in vitro*	-	aortic rings	Δ9-THC	cumulative μM range	perf.	unsp.	vasorelaxation/-constriction
					AEA			CB1r	vasorelaxation
					NADA			CB1r	vasorelaxation
rat	O'Sullivan et al. (2005a)	*in vitro*	-	aortic and mes. rings	Δ9-THC	cumulative μM range	perf.	unsp.	vasorelaxation in smaller branches, Ca^{2+}-influx block; vasoconstriction in sup. mes. art. (stem) at high concentration; antagonism to AEA-vasorelaxation
rat	O'Sullivan et al. (2006)	*in vitro*	-	isolated aorta	Δ9-THC	10μM, 2h incubation	-	unsp.	no effect
				mes. art.	Δ9-THC		-		enhanced endothelium-dependent vasorelaxation in sup. mes. art. and potentiated contraction in resistance vessels
rat	Gardiner et al. (2009)	*in vivo*	consc.	systemic	methan-andamide	0.5-5mg/kg	i.v.	CB1r	CB1r-mediated renal and mesenteric vasoconstriction and hindquarter vasodilation
rat	Tamaki et al. (2012)	*in vitro*	-	mes. art.	AEA	0.1-1nM	perf.	CB1r	vasodilation
						10nM-1μM	perf.	CB1r	vasoconstriction
rat	Wenzel et al. (2013)	*in vitro*	-	isolated perf. lung	AEA	10μM	perf.	CB1r	pulmonary vasoconstriction

Table S2 continued

rat	Iring et al. (2013)	*in vivo*	ur. g.a	cerebral	AM-251	10mg/kg	i.v.	unsp.	no effect on CoBF
					AM-404	10mg/kg	i.v.	unsp.	initial CoBF$\uparrow$, systemic BP$\uparrow$, followed by CoBF$\downarrow$ and BP$\downarrow$
rabbit	Barbosa et al. (1981)	*in vitro*	-	central ear art.	Δ9-THC	15-30mM	perf.	unsp.	sustained contractions by NA release
rabbit	Wahn et al. (2005)	*in vitro*	-	isolated lung	AEA	5μM	perf.	CB1r	pulmonary artery pressure$\uparrow$
					2-AG	0.4μ	perf.	CB1r	pulmonary artery pressure$\uparrow$ (via COX-2 metabolites)
					R-methan-andamide	unsp.	perf.	CB1r	no effect
					Δ9-THC	unsp.	perf.	unsp.	no effect
					HU 210	unsp.	perf.	CB1r	no effect
dog	Cavero et al. (1972)	*in vivo*	s-p g.a.	systemic	Δ9-THC	2.5mg/kg	i.v.	unsp.	BP$\downarrow$, HR$\downarrow$, cardiac output$\downarrow$, splanchnic vasoconstriction
dog	Cavero et al. (1974)	*in vivo*	s-p g.a.	systemic	Δ9-THC	2.5mg/kg	i.v.	unsp.	dose-dependent BP$\downarrow$, HR$\downarrow$, cardiac output$\downarrow$, right ventricular contractile force$\downarrow$, total peripheral vascular resistance$\downarrow$
dog	Jandhyala et al. (1976)	*in vivo*	s-p g.a.	systemic	Δ9-THC	2.5mg/kg	i.v.	unsp.	HR$\downarrow$, pulmonary blood flow$\downarrow$, pulmonary artery pressure$\uparrow$, pulmonary vascular resistance$\uparrow$
pig	Vaddady et al. (2011)	*in vivo*	g.a.	hemor. shock model	Δ9-THC	0.5mg/kg 1mg/kg 4mg/kg	i.v.	unsp.	selective vasoconstriction in liver microvasculature

Abbreviations: Consc. consciousness, Appl. application mode, Targ. target, ur. urethane, g.a. general anesthesia, THC tetrahydrocannabinol, i.v. intravenous, unsp. unspecified, BP blood pressure, HR heart rate, hindqu. hindquarters, i.a. intraarterial, AEA anandamide, CB1r cannabinoid receptor type 1, CB2r cannabinoid receptor type 2, consc. conscious, CBF cerebral blood flow, rCBF regional cerebral blood flow, perf. perfusion, NADA N-arachnidonoyl-dopamine,mes. mesenteric, art. artery, CoBF cerebrocortical blood flow, NA noradrenaline, s-p sodium-pentobarbital, hem. hemorrhagic.

REFERENCES

Adams, M. D., Earnhardt, J. T., Dewey, W. L., and Harris, L. S. (1976). Vasoconstrictor actions of delta8- and delta9-tetrahydrocannabinol in the rat. *J Pharmacol Exp Ther* 196, 649–56. url:jpet.aspetjournals.org

Al Suleimani, Y. M., Al Mahruqi, A. S., and Hiley, C. R. (2015). Mechanisms of vasorelaxation induced by the cannabidiol analogue compound o-1602 in the rat small mesenteric artery. *Eur J Pharmacol* 765, 107–14. doi:10.1016/j.ejphar.2015.08.021

Baranowska-Kuczko, M., Kozłowska, H., Kloza, M., Karpińska, O., Toczek, M., Harasim, E., et al. (2016). Protective role of cannabinoid cb1 receptors and vascular effects of chronic administration of faah inhibitor urb597 in doca-salt hypertensive rats. *Life Sci* 151, 288–299. doi:10.1016/j.lfs.2016.03.014

Barbosa, P. P., Lapa, A. J., Lima-Landman, M. T., and Valle, J. R. (1981). Vasoconstriction induced by delta 9-tetrahydrocannabinol on the perfused rabbit ear artery. *Arch Int Pharmacodyn Ther* 252, 253–61. Abstract retrieved from http://ncbi.nlm.nih.gov (Accession No. 6272661)

Bloom, A. S., Tershner, S., Fuller, S. A., and Stein, E. A. (1997). Cannabinoid-induced alterations in regional cerebral blood flow in the rat. *Pharmacol Biochem Behav* 57, 625–31. doi:10.1016/S0091-3057(96)00475-3

Breyne, J., Van de Voorde, J., and Vanheel, B. (2006). Characterization of the vasorelaxation to methanandamide in rat gastric arteries. *Can J Physiol Pharmacol* 84, 1121–32. doi:10.1139/y06-058

Cavero, I., Buckley, J. P., and Jandhyala, B. S. (1973). Hemodynamic and myocardial effects of (-)-delta9-trans-tetrahydrocannabinol in anesthetized dogs. *Eur J Pharmacol* 24, 243–51. doi:10.1016/0014-2999(73)90078-2

Cavero, I., Ertel, R., Buckley, J. P., and Jandhyala, B. S. (1972). Effects of (-)- 9 -trans-tetrahydrocannabinol on regional blood flow in anesthetized dogs. *Eur J Pharmacol* 20, 373–6. doi:10.1016/0014-2999(72)90201-4

Cavero, I., Lokhandwala, M. F., Buckley, J. P., and Jandhyala, B. S. (1974). The effect of (minus)-delta 9-trans-tetrahydrocannibinol on myocardial contractility and venous return in anesthetized dogs. *Eur J Pharmacol* 29, 74–82. doi:10.1016/0014-2999(74)90172-1

Ellis, E. F., Moore, S. F., and Willoughby, K. A. (1995). Anandamide and delta 9-thc dilation of cerebral arterioles is blocked by indomethacin. *Am J Physiol* 269, H1859–64. doi:10.1152/ajpheart.1995.269.6.H1859

Fleming, I., Schermer, B., Popp, R., and Busse, R. (1999). Inhibition of the production of endothelium-derived hyperpolarizing factor by cannabinoid receptor agonists. *Br J Pharmacol* 126, 949–60. doi:10.1038/sj.bjp.0702381

Gardiner, S. M., March, J. E., Kemp, P. A., and Bennett, T. (2009). Factors influencing the regional haemodynamic responses to methanandamide and anandamide in conscious rats. *Br J Pharmacol* 158, 1143–52. doi:10.1111/j.1476-5381.2009.00363.x

Gebremedhin, D., Lange, A. R., Campbell, W. B., Hillard, C. J., and Harder, D. R. (1999). Cannabinoid cb1 receptor of cat cerebral arterial muscle functions to inhibit l-type ca2+ channel current. *Am J Physiol* 276, H2085–93. doi:10.1152/ajpheart.1999.276.6.H2085

Graham, J. D. and Li, D. M. (1973). Cardiovascular and respiratory effects of cannabis in cat and rat. *Br J Pharmacol* 49, 1–10. doi:10.1111/j.1476-5381.1973.tb08262.x

Grainger, J. and Boachie-Ansah, G. (2001). Anandamide-induced relaxation of sheep coronary arteries: the role of the vascular endothelium, arachidonic acid metabolites and potassium channels. *Br J Pharmacol* 134, 1003–12. doi:10.1038/sj.bjp.0704340

Hillard, C. J., Ho, W.-S., Thompson, J., Gauthier, K. M., Wheelock, C. E., Huang, H., et al. (2007). Inhibition of 2-arachidonoylglycerol catabolism modulates vasoconstriction of rat middle cerebral artery by the thromboxane mimetic, u-46619. *Br J Pharmacol* 152, 691–8. doi:10.1038/sj.bjp.0707468

Ho, W.-S. V. and Gardiner, S. M. (2009). Acute hypertension reveals depressor and vasodilator effects of cannabinoids in conscious rats. *Br J Pharmacol* 156, 94–104. doi:10.1111/j.1476-5381.2008.00034.x

Ho, W.-S. V. and Hiley, C. R. (2003). Endothelium-independent relaxation to cannabinoids in rat-isolated mesenteric artery and role of ca2+ influx. *Br J Pharmacol* 139, 585–97. doi:10.1038/sj.bjp.0705280

Iring, A., Ruisanchez, É., Leszl-Ishiguro, M., Horváth, B., Benkő, R., Lacza, Z., et al. (2013). Role of endocannabinoids and cannabinoid-1 receptors in cerebrocortical blood flow regulation. *PLoS One* 8, e53390. doi:10.1371/journal.pone.0053390

Jandhyala, B. S., Malloy, K. P., and Buckley, J. P. (1976). Effects of acute administration of delta9-tetrahydrocannabinol on pulmonary hemodynamics of anesthetized dogs. *Eur J Pharmacol* 38, 183–7. doi:10.1016/0014-2999(76)90216-8

Kawasaki, H., Watanabe, S., Oishi, R., and Ueki, S. (1980). Effects of delta-9-tetrahydrocannabinol on the cardiovascular system, and pressor and behavioral responses to brain stimulation in rats. *Jpn J Pharmacol* 30, 493–502. doi:10.1254/jjp.30.493

Kosersky, D. S. (1978). Antihypertensive effects of delta9-tetrahydrocannabinol. *Arch Int Pharmacodyn Ther* 233, 76–81. Abstract retrieved from http://ncbi.nlm.nih.gov (Accession No. 686909)

MacIntyre, J., Dong, A., Straiker, A., Zhu, J., Howlett, S. E., Bagher, A., et al. (2014). Cannabinoid and lipid-mediated vasorelaxation in retinal microvasculature. *Eur J Pharmacol* 735, 105–14. doi:10.1016/j.ejphar.2014.03.055

Mair, K. M., Robinson, E., Kane, K. A., Pyne, S., Brett, R. R., Pyne, N. J., et al. (2010). Interaction between anandamide and sphingosine-1-phosphate in mediating vasorelaxation in rat coronary artery. *Br J Pharmacol* 161, 176–92. doi:10.1111/j.1476-5381.2010.00878.x

Niederhoffer, N., Schmid, K., and Szabo, B. (2003). The peripheral sympathetic nervous system is the major target of cannabinoids in eliciting cardiovascular depression. *Naunyn Schmiedebergs Arch Pharmacol* 367, 434–43. doi:10.1007/s00210-003-0755-y

O'Sullivan, S. E., Kendall, D. A., and Randall, M. D. (2005a). The effects of delta9-tetrahydrocannabinol in rat mesenteric vasculature, and its interactions with the endocannabinoid anandamide. *Br J Pharmacol* 145, 514–26. doi:10.1038/sj.bjp.0706218

O'Sullivan, S. E., Kendall, D. A., and Randall, M. D. (2005b). Vascular effects of delta 9-tetrahydrocannabinol (thc), anandamide and n-arachidonoyldopamine (nada) in the rat isolated aorta. *Eur J Pharmacol* 507, 211–21. doi:10.1016/j.ejphar.2004.11.056

O'Sullivan, S. E., Kendall, D. A., and Randall, M. D. (2006). Further characterization of the time-dependent vascular effects of delta9-tetrahydrocannabinol. *J Pharmacol Exp Ther* 317, 428–38. doi:10.1124/jpet.105.095828

O'Sullivan, S. E., Randall, M. D., and Gardiner, S. M. (2007). The in vitro and in vivo cardiovascular effects of delta9-tetrahydrocannabinol in rats made hypertensive by chronic inhibition of nitric-oxide synthase. *J Pharmacol Exp Ther* 321, 663–72. doi:10.1124/jpet.106.116566

O'Sullivan, S. E., Sun, Y., Bennett, A. J., Randall, M. D., and Kendall, D. A. (2009). Time-dependent vascular actions of cannabidiol in the rat aorta. *Eur J Pharmacol* 612, 61–8. doi:10.1016/j.ejphar.2009.03.010

O'Sullivan, S. E., Tarling, E. J., Bennett, A. J., Kendall, D. A., and Randall, M. D. (2005c). Novel time-dependent vascular actions of delta9-tetrahydrocannabinol mediated by peroxisome proliferator-activated receptor gamma. *Biochem Biophys Res Commun* 337, 824–31. doi:10.1016/j.bbrc.2005.09.121

Pakdeechote, P., Dunn, W. R., and Ralevic, V. (2007). Cannabinoids inhibit noradrenergic and purinergic sympathetic cotransmission in the rat isolated mesenteric arterial bed. *Br J Pharmacol* 152, 725–33. doi:10.1038/sj.bjp.0707397

Pratt, P. F., Hillard, C. J., Edgemond, W. S., and Campbell, W. B. (1998). N-arachidonylethanolamide relaxation of bovine coronary artery is not mediated by cb1 cannabinoid receptor. *Am J Physiol* 274, H375–81. doi:10.1152/ajpheart.1998.274.1.H375

Sánchez-Pastor, E., Andrade, F., Sánchez-Pastor, J. M., Elizalde, A., Huerta, M., Virgen-Ortiz, A., et al. (2014). Cannabinoid receptor type 1 activation by arachidonylcyclopropylamide in rat aortic rings causes vasorelaxation involving calcium-activated potassium channel subunit alpha-1 and calcium channel, voltage-dependent, l type, alpha 1c subunit. *Eur J Pharmacol* 729, 100–6. doi:10.1016/j.ejphar.2014.02.016

Siqueira, S. W., Lapa, A. J., and Ribeiro do Valle, J. (1979). The triple effect induced by delta 9-tetrahydrocannabinol on the rat blood pressure. *Eur J Pharmacol* 58, 351–7. doi:10.1016/0014-2999(79)90305-4

Stein, E. A., Fuller, S. A., Edgemond, W. S., and Campbell, W. B. (1998). Selective effects of the endogenous cannabinoid arachidonylethanolamide (anandamide) on regional cerebral blood flow in the rat. *Neuropsychopharmacology* 19, 481–91. doi:10.1016/S0893-133X(98)00043-8

Su, E.-N., Kelly, M. E., Cringle, S. J., and Yu, D.-Y. (2015). Role of endothelium in abnormal cannabidiol-induced vasoactivity in retinal arterioles. *Invest Ophthalmol Vis Sci* 56, 4029–37. doi:10.1167/iovs.14-14879

Tamaki, C., Nawa, H., Takatori, S., Oda, S., Sendo, T., Zamami, Y., et al. (2012). Anandamide induces endothelium-dependent vasoconstriction and cgrpergic nerve-mediated vasodilatation in the rat mesenteric vascular bed. *J Pharmacol Sci* 118, 496–505. doi:10.1254/jphs.11236FP

Vaddady, P. K., Mehrotra, N., Zhang, X., Yates, C. R., Moore, B. M., and Meibohm, B. (2011). Pharmacokinetics of a combination of delta9-tetrahydro-cannabinol and celecoxib in a porcine model of hemorrhagic shock. *Biopharm Drug Dispos* 32, 89–98. doi:10.1002/bdd.740

Varga, K., Lake, K. D., Huangfu, D., Guyenet, P. G., and Kunos, G. (1996). Mechanism of the hypotensive action of anandamide in anesthetized rats. *Hypertension* 28, 682–6. doi:10.1161/01.HYP.28.4.682

Wagner, J. A., Abesser, M., Karcher, J., Laser, M., and Kunos, G. (2005). Coronary vasodilator effects of endogenous cannabinoids in vasopressin-preconstricted unpaced rat isolated hearts. *J Cardiovasc Pharmacol* 46, 348–55. doi:10.1097/01.fjc.0000175437.87283.f2

Wahn, H., Wolf, J., Kram, F., Frantz, S., and Wagner, J. A. (2005). The endocannabinoid arachidonyl ethanolamide (anandamide) increases pulmonary arterial pressure via cyclooxygenase-2 products in isolated rabbit lungs. *Am J Physiol Heart Circ Physiol* 289, H2491–6. doi:10.1152/ajpheart.00718.2005

Wenzel, D., Matthey, M., Bindila, L., Lerner, R., Lutz, B., Zimmer, A., et al. (2013). Endocannabinoid anandamide mediates hypoxic pulmonary vasoconstriction. *Proc Natl Acad Sci U S A* 110, 18710–5. doi:10.1073/pnas.1308130110

Wheal, A. J., Bennett, T., Randall, M. D., and Gardiner, S. M. (2007). Cardiovascular effects of cannabinoids in conscious spontaneously hypertensive rats. *Br J Pharmacol* 152, 717–24. doi:10.1038/sj.bjp.0707410

White, R. and Hiley, C. R. (1998). The actions of some cannabinoid receptor ligands in the rat isolated mesenteric artery. *Br J Pharmacol* 125, 533–41. doi:10.1038/sj.bjp.0702111

Zhang, M., Martin, B. R., Adler, M. W., Razdan, R. K., Ganea, D., and Tuma, R. F. (2008). Modulation of the balance between cannabinoid cb(1) and cb(2) receptor activation during cerebral ischemic/reperfusion injury. *Neuroscience* 152, 753–60. doi:10.1016/j.neuroscience.2008.01.022
